# Analysis of Trends in Resistance to Fluoroquinolones and Extended Spectrum Beta-Lactams among *Salmonella* Typhi Isolates Obtained from Patients at Four Outpatient Clinics in Nairobi County, Kenya

**DOI:** 10.4236/aim.2018.87038

**Published:** 2018-07-30

**Authors:** Susan Mutile Kavai, Mourine Kangogo, Anne W. T. Muigai, Samuel Kariuki

**Affiliations:** 1 Institute of Tropical Medicine and Infectious Diseases, Jomo Kenyatta University of Agriculture and Technology, Nairobi, Kenya; 2 Department of Medical Microbiology, Jomo Kenyatta University of Agriculture and Technology, Nairobi, Kenya; 3 Department of Botany, Jomo Kenyatta University of Agriculture and Technology, Nairobi, Kenya; 4 Centre for Microbiology Research, Kenya Medical Research Institute, Nairobi, Kenya

**Keywords:** Resistance Trends, *Salmonella* Typhi, *β*-Lactams, Flouroquinolones, Nairobi County, Kenya

## Abstract

Typhoid fever caused by the bacterium *Salmonella enterica* serovar Typhi (*S*. Typhi) causes an estimated 25 million illnesses and approximately 200,000 deaths annually mostly in developing countries. Although the management of typhoid fever has been effectively through antibiotic treatment, *S*. Typhi is increasingly becoming resistant to the currently recommended drugs. This study utilized a quasi-experimental design focusing on archived samples to describe antimicrobial susceptibility patterns of *S*. Typhi and determine the genetic basis of resistance to the two most commonly used classes of antimicrobials. A total sample size of 287 isolates of *S*. Typhi isolates stored in −80°C freezer at the Centre for Microbiology Research was utilized. Isolates were subjected to antimicrobial susceptibility testing to commonly available antimicrobials using disk diffusion method, then analyzed for trends in resistance to fluoroquinolones and extended spectrum beta lactams. Among the 287 isolates 158 (55.5%) were found to be Multi Drug Resistant (MDR). This implied that these isolates were resistant to all first line classes of treatment such as ampicillin, chloramphenicol and sulfamethoxazole-trimethroprim. In addition to this, these isolates were also resistant to at least one of the currently recommended drugs of choice, either a *β*-lactam or a fluoroquinolone. This study observed resistances at 18.2% and 15.4% to fluoroquinolones and cephalosporins respectively. PCR results revealed presence of bla_*TEM*_, bla_*INT*_ and bla_*CTX-M*_ genes coding for resistance to *β*-lactams in 80% of the isolates that had combined resistance to *β*-lactams and fluoroquinolones. It is likely that recent heavy use of these classes of antimicrobials is driving resistances to these antimicrobials.

## 1. Introduction

Typhoid fever which is a prolonged bacteremic illness with or without diarrhea [1] is caused by the bacteria *Salmonella enterica* serovar Typhi (*S*. Typhi). *Salmonella* Typhi is primarily adapted to humans as the primary host [2]. Typhoid fever is mainly a disease of adults and older children [3] over ten years of age and is commonly spread through the fecal-oral route via food, drinks, and contaminated water. Human carriers can also contaminate water or food and spread to other people through ingestion of the contaminated materials [4], [5].

There is great variation in the clinical course of typhoid fever and this ranges from fever to marked multi-organ system toxaemia [6]. Generally, Typhoid fever is characterized by abdominal pains, sore throat, malaise, nausea, vomiting, weakness, dizziness, and fever. Chronic infection or complicated typhoid infection also sometimes causes confusion [7]. Patients who develop severe disease thereafter range from 10% – 15% [8].

In endemic regions, lack of nonspecific features such as diarrhoea, vomiting, or predominantly respiratory symptoms may lead to misdiagnosis of typhoid fever. This is because typically it has an incubation period of 10 – 14 days.

It is also mostly associated with prolonged low-grade fever, headache, malaise, myalgia, dry cough, anorexia, and nausea. In sub-Saharan Africa, the incidence of typhoid fever was estimated at 725 cases/100,000 persons in 2010. However, West and Central Africa lacked studies of incidence data [2] [9].

In a single study in Kibera slums in Kenya, among children 2 to 8 years of age, incidence was estimated at 520 per 100,000 person-years of observation [5]. Multidrug resistant clones of *S*. Typhi are a huge challenge in management of the infection in Kenya. Previous studies recorded a prevalence of 77.2% multidrug resistant *S*. Typhi isolates collected from hospitals within Nairobi County over the period 1997–2007 [10]. Currently over a third of *S*. Typhi isolates in many endemic areas are multidrug resistant. They equally have diminished susceptibility to fluoroquinolones, which have been the drugs of choice for MDR cases over the last ten years [3]. Other alternative drugs including extended spectrum *β*-lactams are also becoming less effective with the growing problem of resistance [11]. The MDR *S*. Typhi is a major problem especially in poor informal settlements in Kenya. In these areas, effective alternative antimicrobials are too expensive to be afforded by majority of the population or else unavailable to the general public [5].

## 2. Materials and Methods

### 2.1. Bacterial Isolates

The study utilized a quasi-experimental design analyzing archived isolates from blood and rectal swabs that were obtained from an ongoing surveillance study. The isolates were stored in −80°C freezers located at Centre for Microbiology Research, KEMRI.

A total of 287 isolates were used of which 90 were from Mukurukwa Njenga (MMM), 61 from Mukuru Reuben (MR), 52 from Municipal County Council (MCC), and 84 from Mbagathi District Hospital (MB).

Briefly, the archived vials containing *S*. Typhi isolates were removed from the −80°C freezer and allowed to thaw at room temperature. A loop-full of the isolate was inoculated using the streak plate method on Mac Conkey agar (Oxoid). Incubation was done at 37°C for 24 hours. *S*. Typhi was confirmed by serotyping tests using the slide agglutination technique whereby polyvalent antisera O, followed by antisera 9, 12, Vi and d (Murex Diagnostics, Dartford, UK).

### 2.2. Antimicrobial Susceptibility Testing

Antimicrobial susceptibility testing was performed on the isolates using the Kirby-Bauer disc diffusion technique [12]. The inoculum for susceptibility testing was compared against the McFarland 0.5 turbidity standard. *E. coli* ATCC 25922 and *S. aureus ATCC* 25923 strains were used as the test quality control organisms. Plates were then incubated at 37°C for 18 hours. Zones of inhibitions were measured and the interpretation of results was done according to Clinical Laboratory Standard Institute guidelines [13].

### 2.3. Polymerase Chain Reaction

34 isolates that had combined resistance to *β*-lactams and fluoroquinolones were further subjected to Polymerase Chain Reaction to detect corresponding resistance genes. Briefly, DNA was extracted from the isolates using the boiling method (10 min boiling, followed by centrifugation 3 min at 13,000 xg). PCR reactions ran for the various resistance genes such as the bla genes for the *β*-lactams and PARE genes for the flouroquinolones. The reaction mixture for PCR consisted of pcr beads, 22 μl pcr water, 1 μl of forward and reverse primer and 1 μDNA template. Negative control reaction was used without isolate DNA. Amplification was conducted in 0.2 ml micro centrifuge tube using a programmable Bio-system thermal cycler.

Amplification conditions consisted of 30 cycles of 94°C for 30 s, 55°C for 30 s, and 72°C for 30 s, with a final extension step of 72°C for 10 min for the quinolone primers (Table 1). For the ESSBL genes PCR conditions included 5 min 94°C, 35 cycles of 60 s at 94°C, 45 s at 65°C with a 0.5°C decrease at each cycle, and 60 s at 72°C, final extension at 72°C for 7 min. Annealing temperatures differed as indicated on Table 2.

### 2.4. Data Analysis

Data analysis was done using the SAS software, version 9.3 (SAS Institute). Chi-square test was applied for P-value derivation for analysis of patient Metadata and risk factors associated with Typhoid Fever. (P < 0.05) was considered statistically significant. WHO-net program was used to analyze trend for resistance with data presented in bar graphs.

## 3. Results

### 3.1. Prevalence of MDR in *S*. Typhi

In the 287 *S*. Typhi utilized in this study, the overall prevalence of MDR was 55.5%. Resistance to fluoroquinolones and *β*-lactams was observed at 18.2% and 15.4% respectively. 34 isolates had combined resistance to fluoroquinolones and *β*-lactams.

### 3.2. Selected Meta Data on the Patients with MDR *S*. Typhi

**Figure 1.**
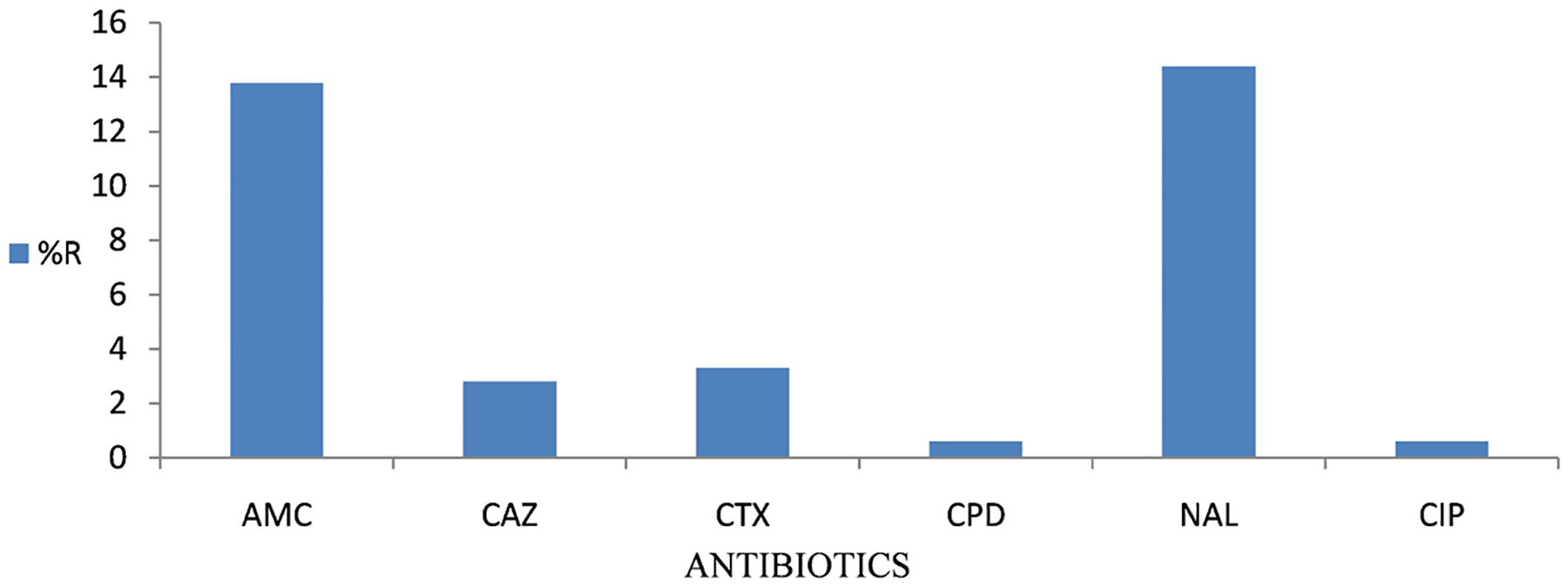
Phenotypic distribution of resistance from isolates obtained from Blood Samples. Resistance levels of isolates obtained from stool samples subjected to phenotypic analysis. The ASTs were against some of the recommended drugs for treatment of Typhoid fever.

**Figure 2.**
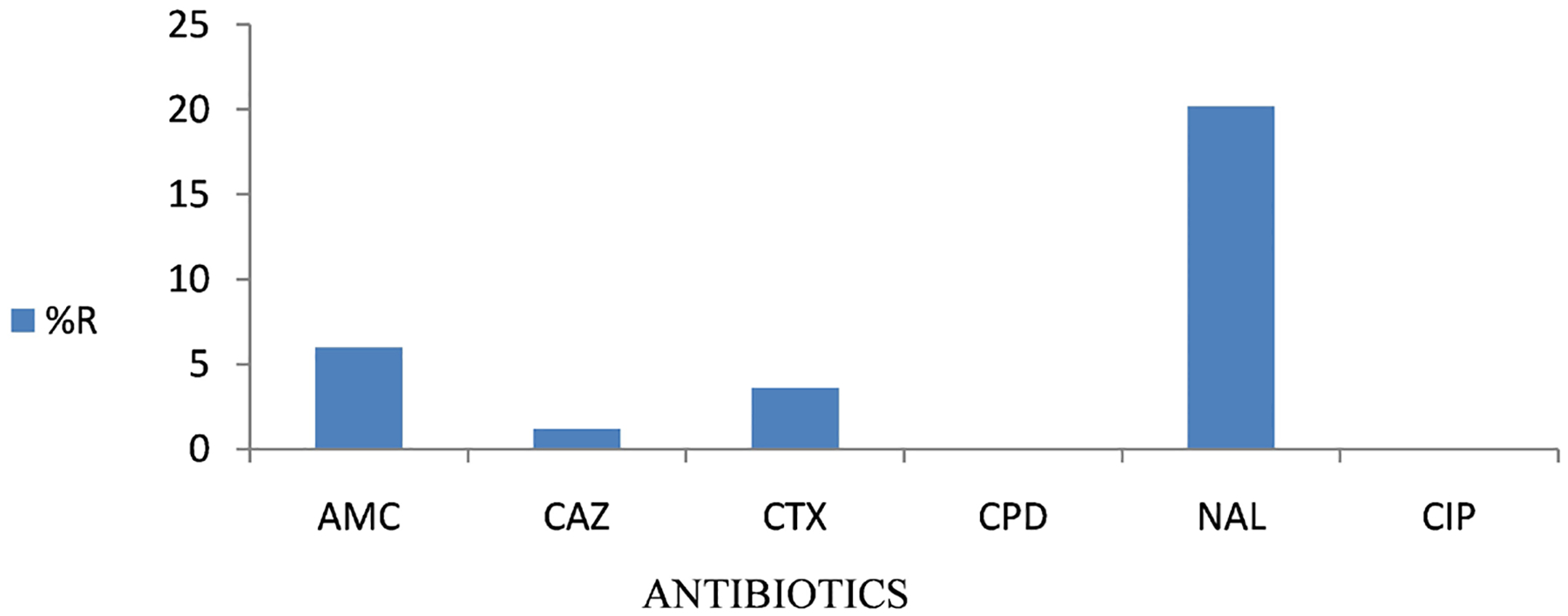
Phenotypic distribution of resistance from isolates obtained from Blood Samples. Resistance levels of isolates obtained from blood samples subjected to phenotypic analysis. The ASTs were against some of the recommended drugs for treatment of Typhoid fever.

A total of 11 (32.4%) of these isolates came from female patients while 23 (67.6%) came from male patients. 21 (61.7%) of the isolates were obtained from stool samples (Figure 1) while 13 (39.3%) were obtained from blood samples (Figure 2). Among this 25 (73.5%) were obtained from patients who had fever at the time they visited the clinic while 9 (26.5%) tested negative for fever.

2 (18.9%) of the female patients who had MDR *S*. Typhi had tested positive for HIV at the time of recruitment to the study while 32 (81.8%) had tested negative. There were more males patients who tested negative for HIV than the females, however there was no male patient who tested positive for HIV in these cohort of patients who had *S*. Typhi that were resistant to the recommended drugs for treatment of Typhoid fever.

A totalof 15 (67.6%) *S*. Typhi obtained from stool samples were MDR, in addition to being MDR, they also had combined resistance to *β*-lactams and fluroquinolones. Most resistance was observed against nalidixic acid (14.4%) and amoxicillin clavulanate (13.8%). Ciprofloxacin and cefpodoxime had the lowest level of resistance at 0.6% each (Figure 1).

Amoxillinclavulanate had the highest resistance level (15.6%) in patients who ≤5 years of age (Table 3). This was closely followed by nalidixic Acid (11.3%). Nalidixic Acid had the highest resistance level (11.3%).

Patients in the bracket of 6 – 10 years had *S*. Typhi that showed highest resistance to nalidixic acid (17.3). This was similar to the patients in the 11 – 16 age brackets (33.3%).

### 3.3. Antimicrobial Profiles for the *S*. Typhi against *β*-Lactams and Fluoroquinolones

**Figure 3.**
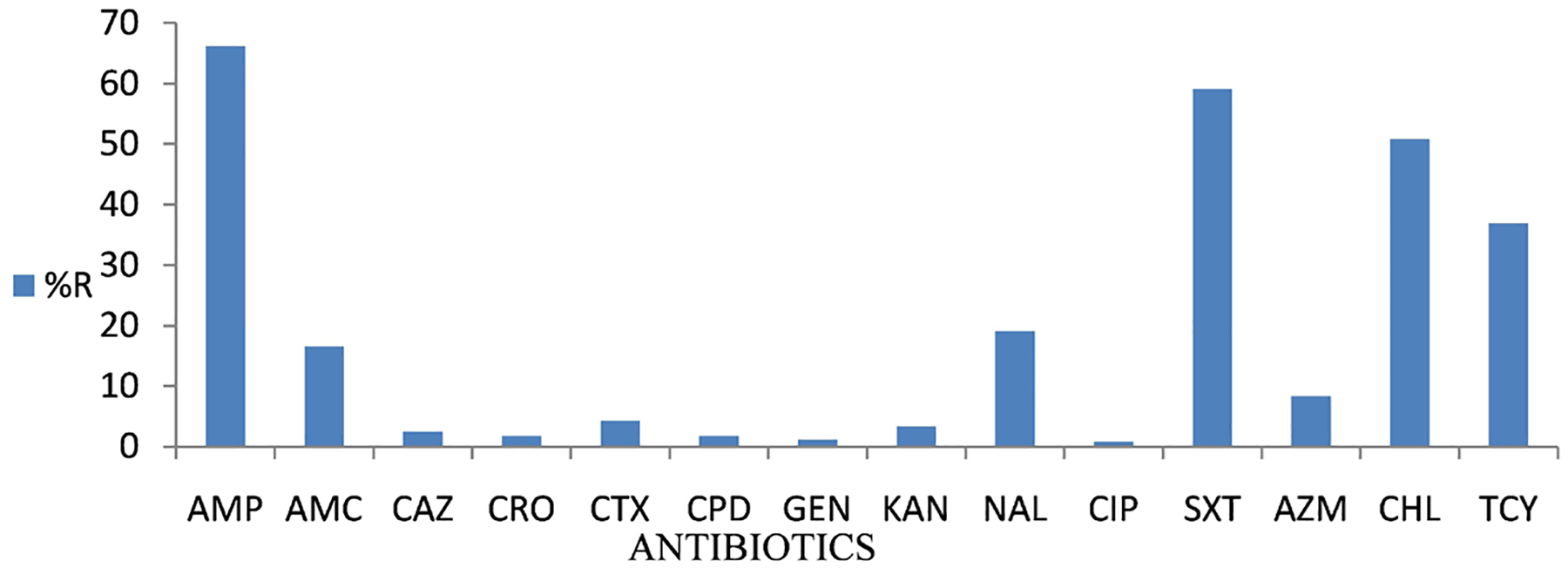
Summary of resistance levels of all the 287 *S*. Typhi subjected to a panel of 14 antibiotics. N = 287 1solates subjected to a panel of 14 selected antibiotics which include Ampicillin (AMP), Amoxicillin Clavulanate (AMC), Ceftazidime (CAZ), Ceftriaxone (CRO), Cefotaxime (CTX), Cefpodoxime (CPD), Gentamicin (CN), Kanamycin (K), Nalidixic Acid (NAL), Ciprofloxacin (CIP), Sulfamethoxazole-trimethroprim (SXT), Azithromycin (AZM), Chloramphenicol (C), and Tetracycline (TCY).

The most common resistant phenotype was ampicillin (64.9%), sulfamethoxazole-trimethroprim (59.6%), and chloramphenicol, (51.7%) (Figure 3). Resistance was also observed against nalidixic Acid (16.2%) and amoxicillin clavaulate (11.3%). cephalosporin’s’ had a low resistance compared to other antimicrobials (Table 4).

There was no significant association between the Samples the *S*. Typhi were obtained from and the gender of the patients the samples came from in relation to contracting Typhoid fever (Table 1). There also was no significant relationship between the gender of the patients and their HIV status in relation to Typhoid fever. There was no significant association between the gender of the patients and their malaria status in relation to Typhoid fever. The P value was > 0.05 indicating that the malaria status of these patients who had resistant strains was not a basis of them having the disease neither was their gender. There was no significant association between the gender of the patients and their Fever status in relation to having Typhoid fever. The P value was >0.05 indicating that the fever status of these patients who had resistant strains was not a basis of them having the disease neither was their gender.

#### PCR results for selected Resistance genes.

**Plate 1.**
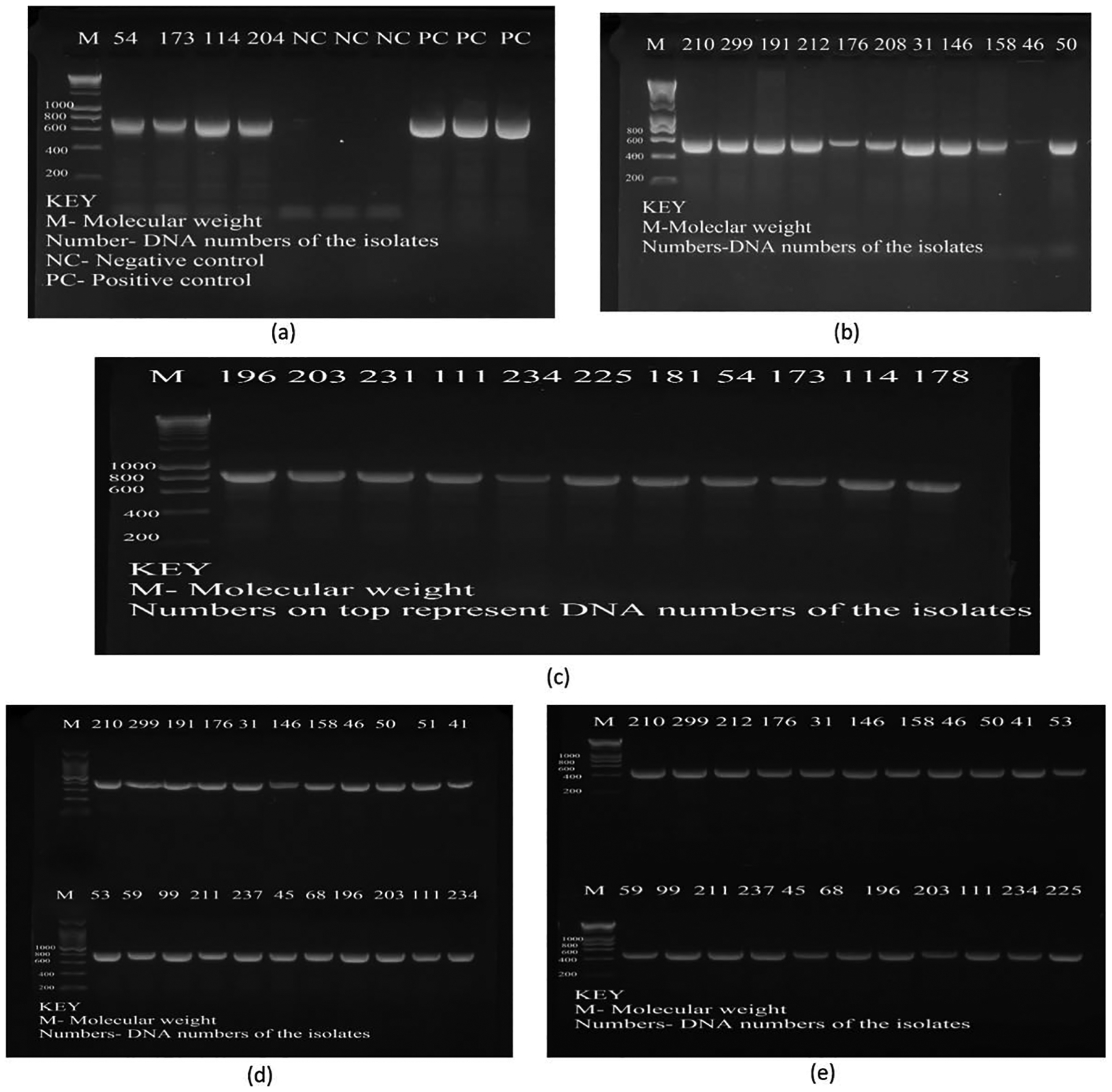
Electrophoresis gel results for blagenes. (a) *bla*_*INT gene*_ 650 bp; (b) *bla*_*CTX-M*_ 593 bp and (c) *bla*_*TEM*_ 865 bp. M-Molecular weight Ladder; NC-Negative Control (Sterile distilled water); PC-Positive Control (known positive control strains). *Numbers at the top represent DNA numbers of the isolate. **Electrophoresis gel results for quinolonegenes. Plates (d) *PARC***_***gene***_ **412 bp; and (e) *PAREgene* 272 bp**. M-Molecular weight Ladder; NC-Negative Control (Sterile distilled water); PC-Positive Control (known positive control strains). *Numbers at the top represent DNA numbers of the isolate.

A total of 27 (80%) isolates subjected to PCR were found to have resistance genes for both *β*-lactams and flouroquinolones. Beta lactamase genes such as *bla*_TEM_, *bla*_CTX-M_ and *bla*_INT_ genes were found to be present in isolates numbers such as 54, 173, 210, 191, 31, 146, 114, 204, 234, 225, 181 and 178 (Plate 1(a), Plate 1(b) and Plate 1(c)). PARE and PARC (Plate 1(d) and Plate 1(e)) genes coding for quinolone resistance were also present for most isolates such as 210, 176, 31, 146, 46, 50, 51, and 299.

## 4. Discussion

The overall prevalence of MDR *S*. Typhi in this study was 55.5%. In 1997–1999 a study done in Kenya from hospital isolates found the MDR in *S*. Typhi to be 65% [14]. While another study carried out in the same study sites in 2010 had the prevalence of MDR *S*. Typhi at 70% [3]. The prevalence in this study clearly shows a decrease in the MDR *S*. Typhi. Variations in prevalence rates over time can be attributed to environmental, economic and social settings of a community especially the ones involved these studies, including different patterns of antimicrobial use for treatment of typhoid and other endemic bacterial diseases over time.

Characterization of outbreaks caused by MDR *S*. Typhi in Asia and some parts of Africa has been well documented [5] [10]. Typhoid fever outbreaks in Sub-Saharan Africa are rarely documented hence data on prevalence and antimicrobial susceptibility trends are scarce [10].

Therefore the observed MDR *S*. Typhi in Kenya though showing a decrease over time is still something to worry about as *S*. Typhi are multidrug resistant to most of the commonly available antimicrobials.

Data from this study confirms there is rise in resistance in the recommended drugs for Typhoid Fever such as nalidixic acid (16.2%) and amoxicillin clavulanate (11.3%). The increased prevalence of MDR *S*. Typhi strains resistant to nalidixic acid should be of public health concern as it is a marker for possible complete fluoroquinolone resistance arising [15].

Resistance was encoded on PARC and PARE genes. In our settings cases of MDR infections are managed using fluoroquinolones, such as ciprofloxacin and norfloxacin. These have been widely used in the last twenty years. Currently, most patients who turned positive for Typhoid fever emerging from slow fever and other symptoms had the clinicians recommend ciprofloxacin as the drug of choice [16].

However with the current MDR prevalence of *S*. Typhi to drugs of choice, it is expected that the resistance to ciprofloxacin would eventually rise if proper measures are not taken to counter resistance [17]. In this study, cephalosporins had a low resistance compared to other antimicrobials. We observed bla_CTX-M_, bla_TEM_, bla_INT_ which encode resistance to extended spectrum beta lactams.

However for the recommended drugs of treatment for typhoid fever, absolutely no resistance should have been observed. This implies that these drugs could still be effective. In cases where there is uncertainty about the susceptibility of a strain or indeed the diagnosis, it is advisable to select an extended spectrum cephalosporin such as ceftriaxone as the safest choice especially if the patient is admitted to hospital as resistance is currently unlikely [18]. It is important to note that resistance to azithromycin which is another effective oral option for uncomplicated typhoid diseasewas less than 10%. In order to secure its continued effectiveness its use should be prudent for management of typhoid disease [19].

Even in settings in the United States, where Typhoid fever is not endemic, patients with similar antibiotic resistance phenotypes show evidence of a longer time to fever clearance and more frequent treatment failure, thus MDR *S*. Typhi is likely to increase overall hospital stay and costs for patients in our settings [20].

## 5. Conclusion

This study observed very high resistance to ampicillin, sulfamethoxazole-trimethroprim, choramphenicol and tetracycline which were the most commonly available antimicrobials in our settings. Resistance was also observed in the *β*-lactams especially amoxicillin clavulanate and nalidixic acid. Emerging resistance to nalidixic acid may be a precursor to low level resistance to flouroquinolones, a potentially worrisome situation as this is the current drug of choice for treatment of typhoid fever in Kenya.

## Figures and Tables

**Table 1.** Primers for PCR amplification and sequencing of genes coding for quinolone resistance in the QRDR and plasmid mediated resistance.

Primer	Primer sequence	Annealing Temps (°C)	Expected Bps
PARC1	5’-ATGAGCGATATGGCAGAGCG-3’	57	412
PARC2	5’-TGACCGAGTTCGCTTAACAG-3’		
PARE1	5’-GACCGAGCTGTTCCTTGTGG-3’	55	272
PARE2	5’-GCGTAACTGCATCGGGTTCA-3’		

**Table 2.** Nucleotide sequences of PCR primers used to amplify ESBL genes.

Primer	Primer sequence	Annealing Temps (°C)	Expected Bps
*bla*_CTX-M_	5’-SCSATGTGCAGYACCAGTAA-3’ 5’-CCGCRATATGRTTGGTGGTG-3’	60	593
*bla*_INT_	5’-GCCCAKCCGACGAACCAGC-3’ 5’-ACCTTCAAGATCCCSCTSGC-3’	50	650
*bla*_TEM_	5’-TCGGGGAAATGTGCGCG-3’ 5’-TGCTTAATCAGTGAGGCACC-3’	55	865

**Table 3.** Resistance levels against various age distributions.

Age	*β*-Lactams				Fluoroquinolones		
	CAZ	CPD	CTX	CRO	AMC	NAL	CIP
≤5 Years	3.5	0.7	2.1	1.4	15.6	11.3	0.7
6 – 10 years	1	0	4.1	1	8.2	17.3	0
11 – 16 years	0	0	6.7	0	3.3	33.3	0

Resistance levels of MDR *S*. Typhi against recommended drugs of treatment for various age distributions. Ceftazidime (CAZ), Cefpodoxime (CPD), Cefotaxime (CTX), Ceftriaxone(CRO), Amoxicillin Clavulanate (AMC), Nalidixic Acid (NAL), Ciprofloxacin (CIP).

**Table 4.** Test of association between genders of the Patients and *S*. Typhi that were resistant to *β*-lactams and Fluoroquinolones.

N = 34	Chi square			Odds Ratio for Gender (Male/Female)		
Variables	Value P = 0.05	Degrees of Freedom	Asymptotic Significance (2-sided)	Value P = 0.05	Upper 95% CI	Lower 95% CI
Gender vs. Sample	0.359a	1	0.549	0.640	0.148	2.768
Gender vs. HIV Status	4.443a	1	0.035	0.818	0.619	1.081
Gender vs. Malaria status	0.451a	1	0.970	4.86	0.100	2.356
Gender vs. History of Fever	0.818a	1	0.366	0.486	0.100	2.356

**Key: N: Number of valid Cases. CI: Confidence Interval**. Tests of associations for various Meta data against gender of the patients who had combined resistance to some of the recommended drugs of choice for treatment of Typhoid fever.
